# The Influence of Viewing Time and Color on Architectural Aesthetic Judgment

**DOI:** 10.3389/fpsyg.2021.752996

**Published:** 2022-01-27

**Authors:** Anbang Dai, Junru Wang, Jie Yu, Hiroatsu Fukuda

**Affiliations:** ^1^Department of Architecture, The University of Kitakyushu, Kitakyushu, Japan; ^2^Department of Neurology and Brain Medical Center, First Affiliated Hospital, School of Medicine, Zhejiang University, Hangzhou, China

**Keywords:** neuroaesthetic, visual perception, cognition, architecture, viewing time

## Abstract

Understanding the factors influencing the aesthetic experience of architectures is an important topic in empirical aesthetics. In this study, we examined the effect of three architectural factors, i.e., ceiling height, openness, and contour, on viewers’ aesthetic appreciation through a series of experiments. In previous studies on architectural aesthetics, participants were usually asked to view an image of an architectural space for a few seconds. The long viewing time allows them to focus on different parts of the architecture and then make an aesthetic judgment. The long viewing time, however, also makes it difficult to obtain viewers’ aesthetic scores for a large number of architectural spaces in a short period. In this study, we shortened the visual presentation time to 200 ms, which allowed the viewers to have only one fixation on the image, and asked the viewers to make an aesthetic judgment. It was found that the experiment with a 200-ms viewing time could establish how the three architectural factors influenced aesthetic judgment as well as previous experiments with a 3,000-ms viewing time, suggesting that aesthetic judgment could be made within one fixation. Additionally, we investigated the impact of color on architectural aesthetic judgment by presenting grayscale images. We found that the three architectural factors influenced aesthetic judgment in similar ways for both color and grayscale images. In summary, we found that color was not a main factor modulating viewers’ architectural aesthetic judgments, and we also presented a way to quickly obtain aesthetic scores for architectural spaces.

## Introduction

What factors drive our aesthetic experience is a topic that receives a considerable amount of attention. The field of empirical aesthetics emphasizes that aesthetic experience should be empirically studied and experimentally validated. Using the empirical aesthetics approach, it has been shown that the relationship between complexity and novelty, and aesthetic appreciation has an inverted U shape – People generally prefer an intermediate level of complexity and novelty (Berlyne, 1973). Empirical aesthetics studies have revealed that the evaluation or production of beauty, ugliness, prettiness, harmony, elegance, shapeliness or charm is governed by a host of factors, such as stimulus symmetry, complexity, novelty, familiarity, artistic style, appeal to social status, and individual preferences (Jacobsen, 2006).

Here, we focus on aesthetic judgment of architectural inner space. Understanding people’s preference for architectural features allows architects to better design structures that satisfy the aesthetic needs of the public. A good architectural design will potentially enhance the user’s comfort, cognition and creativity (Sternberg and Wilson, 2006). Vegetation, stylistic uniformity, homogeneity, and symmetry can influence aesthetic experience and judgment (Jacobsen, 2006; Weber et al., 2008). Contour is also a very important feature of architectural space. A series of studies have shown that most people believe that curvilinear contours are more beautiful than rectilinear contours (Leder and Carbon, 2005; Bar and Neta, 2006; Dazkir and Read, 2011; Vartanian et al., 2013). Ceiling height and openness also affect people’s cognition and emotion in an architectural space (Franz et al., 2005; Meyers-Levy et al., 2007): Under a high ceiling, people tend to have more positive emotions, such as “happy,” “comfortable” and “fun.” Similarly, they tend to experience more positive emotions in spacious architectural spaces than in tight architectural spaces (Stamps, 2010, 2011).

Previous studies have established that ceiling height, openness and contour can affect aesthetic judgment of architectural space. In these studies, however, participants were usually asked to view an image of an architectural space for a few seconds. The long viewing time allowed participants to focus on different parts of the architectural space before making an aesthetic judgment. The long viewing time, however, also makes it difficult to obtain viewers’ aesthetic judgment for a large number of architectural spaces in a short period. In this study, we shortened the viewing time to 200 ms, which allowed the viewers to have only one fixation on the architectural space. Furthermore, we tested whether color, a salient visual feature, can influence how architectural features modulated aesthetic experience.

## Materials and Methods

### Participants

The participants in this study were students at Zhejiang University. They were all right-handed and had no visual impairment or colorblindness. All participants had normal or corrected-to-normal vision and no history of psychosis or neuropathy. The experimental procedure was approved by the Research Ethics Committee of Zhejiang University School of Medicine. All participants signed written informed consent forms before the experiment and received a monetary reward after completing the experiment. A total of 42 participants were recruited. For the condition presenting colorful images, 21 participants were recruited, including 15 males (aged 23.13 ± 2.00 years) and 6 females (aged 23.33 ± 2.25 years). For the condition presenting grayscale images, 21 participants were recruited, including 9 males (aged 22.89 ± 1.90 years) and 12 females (aged 22.25 ± 1.60 years).

### Stimuli and Procedures

**FIGURE 1:**
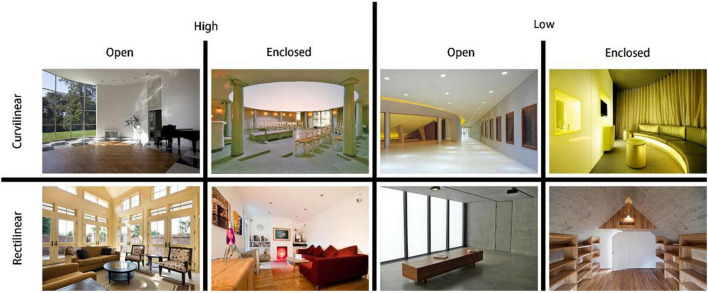
Example of the stimulus. A total of 200 pictures were divided into 8 groups with 25 pictures in each group.

The current study adopted the same set of stimulus used in reference (Vartanian et al., 2013), which had been frequently used to study aesthetic judgment of architectural space (Vartanian et al., 2013, 2015, 2019; Coburn et al., 2020; Skov et al., 2021). The set of stimulus comprised 200 photos of architectural spaces of different styles. Each photo was categorized by three factors, i.e., ceiling height, openness and contour. Each factor had two levels, i.e., high and low for ceiling, open and enclosed for openness, curvilinear and rectilinear for contour. The categorization of each photo was confirmed by two researchers in the field of architectural aesthetics, i.e., two of the authors of reference (Vartanian et al., 2013). The combination of the factors at the various levels generated eight sets of images of spaces with different styles, and each set contained 25 photos (Figure 1).

The experiments were performed in a soundproof room. The participants sat in a chair and rest his or her chin on a chin rest. The height of the chair was adjusted to ensure that the line of sight was at approximately the upper quarter of the screen of the monitor. The experiment stimuli were presented on a 23-inch monitor (Dell E2316H, resolution: 1920 × 1080, refresh rate: 60 Hz, size: 509 × 286 mm). The eye distance from the screen was approximately 900 mm. The view angle of the images were roughly 14 × 9 degree.

**FIGURE 2:**
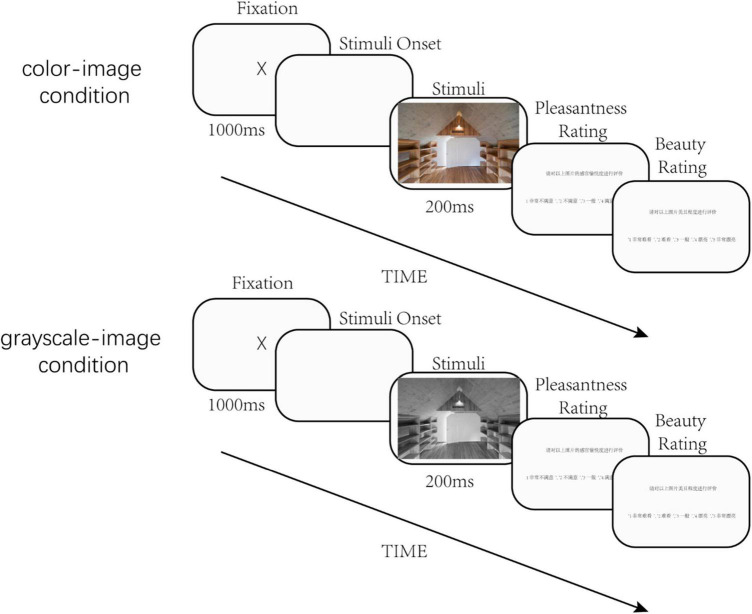
Illustration of the experimental procedure.

The study consisted of two conditions. Each condition included 200 trials, and 1 image was presented in each trial. The steps for each trial were as follows: First, a fixation cross was presented at the center of the screen for 1000 ms. Then, a randomly selected stimulus image was presented for 200 ms, which was followed by two questions asking the participant to score the image in terms of pleasantness and beauty (1 = very unpleasant/ugly; 5 = very pleasant/beautiful). One condition presented the original color images in reference (Vartanian et al., 2013), and the other condition presented the grayscale version of the images. Each RGB color image was converted into a grayscale image by averaging the three color channels (Figure 2).

## Results

### Aesthetic Judgment Under Short Viewing Time

**FIGURE 3:**
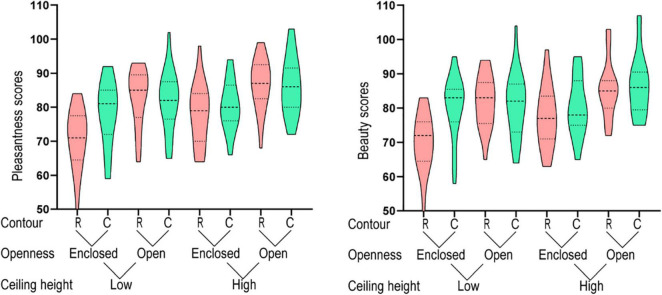
Results for the color-image condition.

In the color-image condition, a three-way repeated measures ANOVA analysis (ceiling height × openness × contour) showed that the main effects of the three factors, i.e., ceiling height, openness and contour, were all statistically significant (Table 1). Overall, structures with high ceilings, open space or curvilinear contours were rated as being more pleasant and beautiful (Figure 3). These results were consistent with previous studies that employed 3,000-ms viewing time (Vartanian et al., 2013, 2015, 2019; Coburn et al., 2020; Skov et al., 2021).

**TABLE 1:** ANOVA analyses of the beauty ratings for the short viewing time condition and the grayscale condition.

Factors	Color condition		Grayscale condition	
		
	Pleasure *P(F)*	Beauty *P(F)*	Pleasure *P(F)*	Beauty *P(F)*
Ceiling height	<0.001(21.39)*	<0.001(17.95)*	<0.001(50.51)*	<0.001(48.83)*
Degree of openness	<0.001(75.11)*	<0.001(62.18)*	<0.001(81.39)*	<0.001(69.31)*
Contour type	0.009(8.32)*	<0.001(20.25)*	0.018(6.64)*	<0.001(16.97)*
Ceiling height × Degree of openness	0.639(0.23)	0.689(0.17)	0.757(0.1)	0.627(0.24)
Ceiling height × Contour type	0.11(2.8)	0.023(6.01)*	<0.001(32.4)*	<0.001(26.53)*
Degree of openness × Contour type	<0.001(27.15)*	<0.001(21.15)*	<0.001(23.36)*	<0.001(27.45)*
Ceiling height × Degree of openness × Contour type	0.035(5.14)*	<0.001(23.26)*	<0.001(19.78)*	<0.001(40.12)*

*p < 0.05. The main effect of each factor is significant, and the rating is higher for higher ceilings, more open space, and curvilinear contours.

### Aesthetic Judgment of Grayscale Images

**FIGURE 4:**
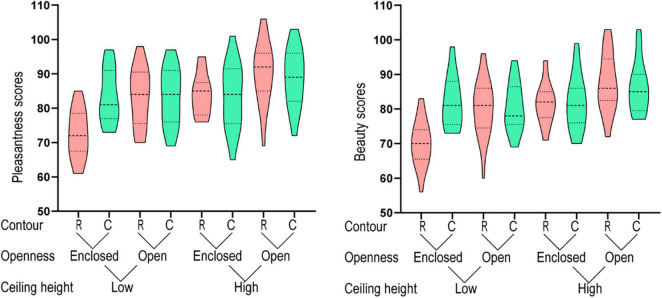
Results for the grayscale-image condition.

The grayscale-image condition was the same as the color-image condition, except that the images were converted into grayscale. A three-way repeated measures ANOVA analysis (ceiling height × openness × contour) showed that the main effects of the three factors, i.e., ceiling height, openness and contour, were statistically significant (Table 1). Structures with high ceilings, open space, or curvilinear contours generated a higher level of pleasantness in the viewer than images of structures with low ceilings, enclosed space, or rectilinear contours (Figure 4). The results in the grayscale-image condition was consistent with the results in the color-image condition, suggesting that color does not significant affected aesthetic judgment of architectural space.

## Discussion

In this study, we find that when viewing images of architectural space for only 200 ms, observers prefer space with higher ceilings, higher degree of openness, and curvilinear contours, compared with space with lower ceilings, lower degree of openness, and rectilinear contours. These conclusions hold for both color and grayscale images of architectural space, and are consistent with previous studies in which aesthetic ratings were collected for images presented for a much longer time, i.e., 3000 ms.

Viewing time can strongly influence perception (Fei-Fei et al., 2007; Mullin et al., 2017). Furthermore, previous study shows that participants recognize normal color images faster than grayscale images (Oliva and Schyns, 2000), indicating that color plays an important role in the early stages of visual processing (Spence et al., 2006) and aids the human cognitive system in rapid recognition (Gegenfurtner and Rieger, 2000; Castelhano and Henderson, 2008). Color, however, is a feature that can be dissociated from architectural features such as ceiling height, openness, and contour, and therefore it is important to test whether the conclusions of previous studies on color images can generalize to grayscale images. Previous studies using color images have shown that observers prefer high ceilings, open space, and curvilinear contours (Vartanian et al., 2013, 2015, 2019; Coburn et al., 2020; Skov et al., 2021), and the current study show that observers prefer the same set of features when viewing grayscale images.

In sum, here we demonstrated that ceiling height, openness, and contour can reliably influence the viewers’ aesthetic judgment, despite of changes in the stimulus duration and color. The results suggest that future studies can collect aesthetic ratings of architectural spaces in a more effective way.

## Data Availability Statement

The raw data supporting the conclusions of this article will be made available by the authors, without undue reservation.

## Ethics Statement

The studies involving human participants were reviewed and approved by Research Ethics Committee of Zhejiang University School of Medicine. The patients/participants provided their written informed consent to participate in this study.

## Author Contributions

AD and HF conceived the experiments and wrote the manuscript. AD and JY performed the experiments. JW analyzed the data. All authors edited the manuscript.

## Conflict of Interest

The authors declare that the research was conducted in the absence of any commercial or financial relationships that could be construed as a potential conflict of interest.

## Publisher’s Note

All claims expressed in this article are solely those of the authors and do not necessarily represent those of their affiliated organizations, or those of the publisher, the editors and the reviewers. Any product that may be evaluated in this article, or claim that may be made by its manufacturer, is not guaranteed or endorsed by the publisher.
