# Supplementary figures and images for: The Influence of Viewing Time and Color on Architectural Aesthetic Judgment
Source: Front Psychol. 2022 Jan 27;12:752996. doi: 10.3389/fpsyg.2021.752996 (PMC8828540; doi:10.3389/fpsyg.2021.752996)

S1.Figure


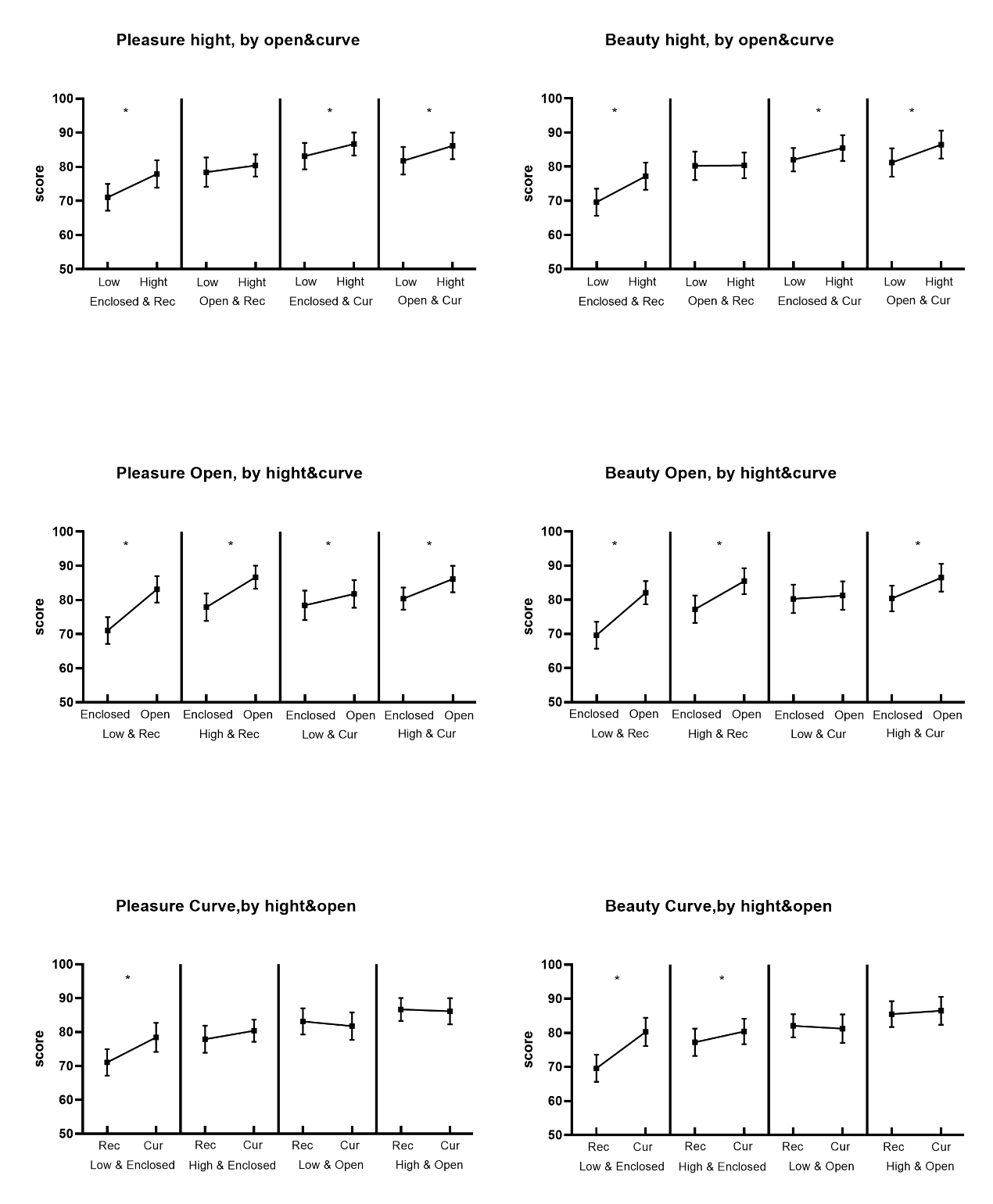


S2.Figure


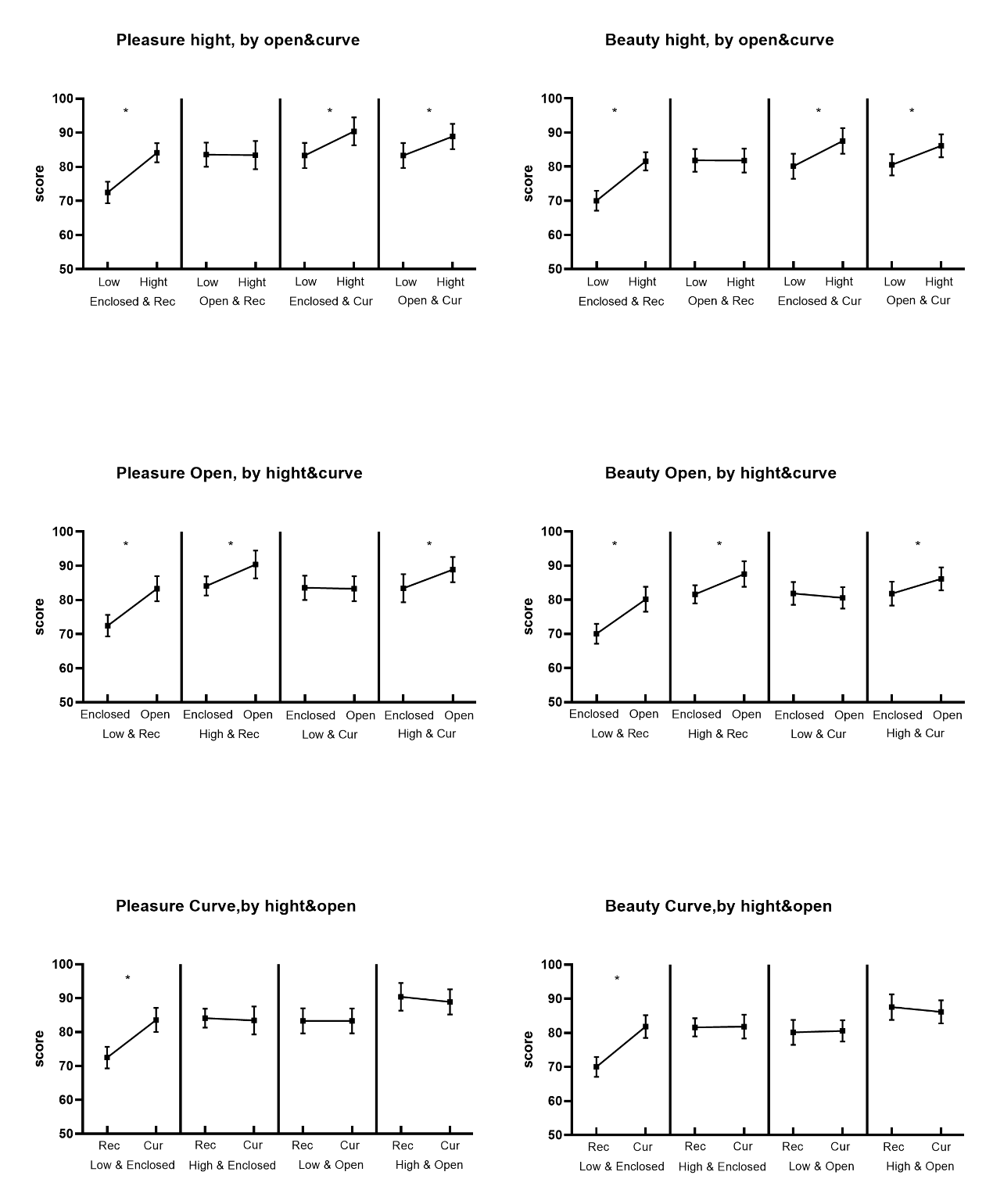

Supplement: Supplementary file 1 [file Data_Sheet_1.docx]
